# The Smac mimetic BV6 cooperates with STING to induce necroptosis in apoptosis-resistant pancreatic carcinoma cells

**DOI:** 10.1038/s41419-021-04014-x

**Published:** 2021-08-30

**Authors:** Sabine Hannes, Rebekka Karlowitz, Sjoerd J. L. van Wijk

**Affiliations:** 1 Institute for Experimental Cancer Research in Pediatrics, Goethe-University, Komturstrasse 3a, 60528 Frankfurt, Germany; 2 General and Visceral Surgery, Goethe-University, Frankfurt, Germany

**Keywords:** Cancer, Cancer

## Abstract

Pancreatic cancer (PC) still remains a major cause of cancer-related death worldwide and alternative treatments are urgently required. A common problem of PC is the development of resistance against apoptosis that limits therapeutic success. Here we demonstrate that the prototypical Smac mimetic BV6 cooperates with the stimulator of interferon (IFN) genes (STING) ligand 2′,3′-cyclic guanosine monophosphate–adenosine monophosphate (2′3′-cGAMP) to trigger necroptosis in apoptosis-deficient PC cells. Pharmacological inhibition of key components of necroptosis signaling, such as receptor-interacting protein 1 (RIPK1), RIPK3, and mixed lineage kinase domain-like protein (MLKL), significantly rescues PC cells from 2′3′-cGAMP/BV6/zVAD.fmk-mediated cell death, suggesting the induction of necroptosis. Consistently, 2′3′-cGAMP/BV6 co-treatment promotes phosphorylation of MLKL. Furthermore, we show that 2′3′-cGAMP stimulates the production of type I IFNs, which cooperate with BV6 to trigger necroptosis in apoptosis-deficient settings. STING silencing via siRNA or CRISPR/Cas9-mediated gene knockout protects PC cells from 2′3′-cGAMP/BV6/zVAD.fmk-mediated cell death. Interestingly, we demonstrate that nuclear factor-κB (NF-κB), tumor necrosis factor-α (TNFα), and IFN-regulatory factor 1 (IRF1) signaling are involved in triggering 2′3′-cGAMP/BV6/zVAD.fmk-induced necroptosis. In conclusion, we show that activated STING and BV6 act together to exert antitumor effects on PC cells with important implications for the design of new PC treatment concepts.

## Introduction

Pancreatic cancer (PC) is a devastating disease with a dismal 5-year survival rate of only 9% [1]. PC is the fourth leading cause of cancer-related death in the United States and the seventh worldwide [1, 2]; despite continuous efforts to improve PC survival, mortality rates have been rising over the last 4 years [1]. As current treatment strategies against PC offer only very limited benefits, there is an urgent medical need for novel therapeutic approaches.

Unfortunately, PC rapidly acquire resistance against apoptosis upon exposure of chemotherapeutic agents [3, 4]. Therefore, induction of necroptosis might be a promising strategy to trigger programmed cell death and to overcome treatment resistance in apoptosis-resistant PC cells [5]. Necroptosis is an alternative form of programmed cell death, which is morphologically and mechanistically distinct from apoptotic cell death [6]. In the absence, or upon pharmacological inhibition, of caspases, necroptosis occurs after formation of the necrosome through coordinated interactions and phosphorylation of RIPK1, RIPK3, and the pseudokinase MLKL, inducing MLKL oligomerization and plasma membrane disruption [7, 8]. Necrosome formation is facilitated by deubiquitination of RIPK1, e.g., upon exposure to second mitochondria-derived activator of caspases (Smac) mimetics, such as BV6, which antagonize cellular inhibitor of apoptosis (cIAP) proteins, which ubiquitinate RIPK1 [9]. The family of IAP proteins is well-known to prevent apoptotic cell death and to modulate nuclear factor-κB (NF-κB) signaling via ubiquitination [10]. Importantly, increased IAP expression has been detected in many cancer types, including PC, and is associated with a poor prognosis [11, 12]. Therefore, synthetic Smac mimetics, which induce cIAP autoubiquitination and proteasomal degradation, are considered promising targeted therapeutics and the Smac mimetics Birinapant and LCL161 are currently being evaluated in clinical phase II studies (https://clinicaltrials.gov/). The mechanisms of necroptosis induction by the prototypical Smac mimetic BV6 and death receptor agonists, such as tumor necrosis factor-α (TNFα) and TNFα-related apoptosis-inducing ligand, are well-studied, but alternative necroptotic stimuli have been identified as well [13–15].

Stimulator of interferon (IFN) genes (STING) is a central regulator of IFN and NF-κB responses, and has emerged as a promising target in cancer immunotherapy [16]. Although many studies investigated STING activity in the tumor microenvironment, there is increasing evidence of intrinsic activation of the cyclic guanosine monophosphate–adenosine monophosphate (GMP–AMP) synthase (cGAS)-STING pathway in cancer cells, suggesting an important role in cellular senescence and tumor development [17, 18]. The carcinogenic influence of STING signaling is cancer-type specific and context-dependent [19, 20], and a dual role of STING in antitumor immunity and tumor promotion has been reported [17, 19, 21, 22]. STING is predominantly activated by 2′,3′-cyclic GMP–AMP (2′3′-cGAMP), a second messenger cyclic dinucleotide, which is generated endogenously by cGAS from adenosine 5′-trisphosphate (ATP) and guanosine-5′-triphosphate upon sensing double-stranded DNA in the cytosol [17, 19]. Activated STING, complexed with TANK-binding kinase 1 (TBK1), translocates from the endoplasmic reticulum to perinuclear regions in an autophagy-resembling process [23]. There, TBK1 induces IFN-regulatory factor 3 phosphorylation and NF-ĸB activation [24], initiating the production of type I IFNs and additional inflammatory cytokines that mediate host immune defenses and antitumor immunity [17]. We, and others, have previously demonstrated a synergistic interaction of BV6 and other Smac mimetics with immunostimulatory cytokines, such as type I and II IFNs, to cooperatively induce cell death in a variety of cancer cell lines [15, 25–27]. Here we show that STING activation by 2′3′-cGAMP combined with BV6 provides a mechanism to trigger necroptotic cell death in apoptosis-deficient PC cells.

## Materials and methods

### Cell lines, chemicals, and antibodies

PC cell lines were kindly provided by D. Saur (Munich, Germany) and maintained in RPMI 1640 or Dulbecco’s modified Eagle medium (Life Technologies, Inc., Eggenstein, Germany), supplemented with 10% or 20% fetal calf serum (Biochrom, Berlin, Germany), 1 mM sodium pyruvate (Life Technologies), and 1% penicillin/streptomycin (Life Technologies) at 37 °C and 5% CO_2_. Cell lines were authenticated by short tandem repeat profiling and were regularly tested for mycoplasma infection.

The X-linked IAP-, cIAP1-, and cIAP2-antagonizing Smac mimetic BV6 [10] was kindly provided by Genentech, Inc. (South San Francisco, CA, USA). Enbrel and ATP disodium salt hydrate were purchased from Sigma-Aldrich (Taufkirchen, Germany); 2′3′-cGAMP from InvivoGen (Toulouse, France); Z-Val-Ala-DL-Asp(OMe)-fluoromethylketone (zVAD.fmk) was obtained from Bachem (Heidelberg, Germany); IFNβ, IFNγ, Necrostatin-1 (Nec-1s), Necrosulfonamide (NSA), and GSK’872 (GSK) from Merck Millipore (Darmstadt, Germany); and Dabrafenib from Selleckchem (Houston, TX, USA). All other chemicals were purchased from Sigma-Aldrich (Taufkirchen, Germany) or Carl Roth (Karlsruhe, Germany), unless indicated otherwise. Cells were pre-incubated with zVAD.fmk, Nec-1s, NSA, or GSK’872 1 h prior to BV6 treatment.

Cell lysates were prepared using RIPA buffer (20 mM Tris HCl pH 8, 150 mM NaCl, 1% Nonidet P-40, 150 mM MgCl_2_, 0,5% sodium deoxycholate) supplemented with 10% SDS, 100 mM sodium orthovanadate, 500 mM sodium fluoride, 100 mM β-glycerolphosphate, 100 mM phenylmethylsulfonyl fluoride, 1 mM protease inhibitor cocktail, and 1 µl/ml Pierce Universal Nuclease (Thermo Fisher Scientific).

The following antibodies were used in this study: rabbit anti-STING (13647S) (Cell Signaling, Beverly, MA, USA), rabbit anti-phospho-STAT1 (9167L) (Cell Signaling), rabbit anti-MLKL (14993S) (Cell Signaling), rabbit anti-phospho-MLKL (ab196436) (Cell Signaling), rabbit anti-caspase-8 (ab227439) (Abcam), rabbit anti-p65 (sc-372X) (Santa Cruz Biotechnologies, Santa Cruz, CA, USA), rabbit anti-phospho-p65 (3033S) (Cell Signaling), rabbit anti-IκBα (9242S) (Cell Signaling), mouse anti-phospho-IκBα (9246S) (Cell Signaling), rabbit anti-NF-κB-inducing kinase (4994S) (NIK) (Cell Signaling), rabbit anti-phospho-p100 (4810L) (Cell Signaling), mouse anti-p52 (05–361) (Merck Millipore), rabbit anti-TBK1 (ab40676) (Abcam), rabbit anti-phospho-TBK1 (ab109272) (Abcam), mouse anti-IRF1 (sc-137061) (Santa Cruz Biotechnologies), mouse anti-β-Actin (A5441) (Sigma-Aldrich), and anti-glyceraldehyde 3-phosphate dehydrogenase (GAPDH) (5G4cc) (HyTest, Turku, Finland). Secondary antibodies conjugated to horseradish peroxidase (Santa Cruz Biotechnology) and enhanced chemiluminescence were used for detection (Amersham Bioscience, Freiburg, Germany). Alternatively, secondary antibodies labeled with IRDye infrared dyes were used for fluorescence detection (Odyssey Imaging System, LI-COR Bioscience, Bad Homburg, Germany). All western blottings shown are representative of at least two independent experiments.

### 2′3′-cGAMP stimulation

To stimulate STING, the indicated cell lines were subjected to permeabilization with digitonin buffer [50 mM HEPES (Life Technologies, Inc.), 100 mM KCl, 3 mM MgCl_2_, 0.1 mM dithiothreitol, 85 mM sucrose, 0.2% bovine serum albumin, 1 mM ATP, digitonin (AsPc-1: 5 µg/ml; BxPc-3 and DanG: 10 ng/ml; Capan-1: 10 µg/ml)], pH 7 containing 2′3′-cGAMP (4 µg/ml) at 37 °C. Medium was replaced after 10 min.

### Quantification of cell death

The amount of cell death was quantified by fluorescence-based microscopic analysis of propidium iodide (PI) uptake using Hoechst 33342 (Sigma-Aldrich, 14533) and PI (Sigma-Aldrich, P4864) double staining, to determine plasma membrane permeability. For this, either flow cytometry (FACS Canto II, BD Biosciences) or high-content imaging with the ImageXpress Micro XLS Widefield High-Content Analysis System (Molecular Devices, LLC, Biberach an der Riss, Germany) and the ImageXpress Micro XLS and MetaXpress Software (Molecular Devices Sunnyvale, CA, USA) were used according to the manufacturer’s instructions.

### Quantitative real-time PCR

To analyze changes in gene expression, total RNA was isolated from the indicated cell lines using the peqGOLD Total RNA kit (Peqlab, Erlangen, Germany) according to the manufacturer’s instructions. Synthesis of cDNA was performed with 1 μg of total RNA using the RevertAid H Minus First Strand cDNA Synthesis Kit (MBI Fermentas GmbH, St. Leon-Rot, Germany) according to the manufacturer’s protocol with the use of random primers. Gene expression levels were quantified using SYBR green-based quantitative real-time PCR (Applied Biosystems, Darmstadt, Germany), using the 7900GR fast real-time PCR system (Applied Biosystems). Expression levels of TNFα mRNA were assessed using the TaqMan Gene Expression Assay (Life Technologies, Darmstadt, Germany) according to the manufacturer’s protocol. Data were normalized to 28S-rRNA expression and relative target transcript expression levels were calculated compared to the reference transcript using the ΔΔCT method [28]. At least three independent experiments in duplicates are shown. All primers were purchased at Eurofins (Hamburg, Germany) (Supplementary Table 1).

### RNAi-mediated gene silencing

Transient knockdown was achieved using reverse-transfected small interfering RNA (siRNA, 20 nM, Silencer Select, Thermo Scientific) against STING (#1: S5064, #2: S50646), p65/RelA (#1: s11914, #2: s11915), and IRF1 (#1: s7501, #2: s7502) or non-targeting control siRNA (4390843) for 48 h using Lipofectamine RNAi MAX (Invitrogen) and OptiMEM (Life Technologies, Inc.).

### Generation of CRISPR/Cas9 KO cells

CRISPR/Cas9 non-human target (nHT), STING, and caspase-8 knockout (KO) cell lines were generated as described previously [29]. Briefly, three independent guide RNAs (gRNAs) were designed with 5′- and 3′-*BsmB1* restriction site overhangs (Supplementary Table 2), annealed, and ligated into pLenti-CRISPRv2 (Addgene plasmid #52961). Lentiviral particles were generated by co-transfecting pLenti-CRISPRv2 STING or caspase-8 gRNAs with pPAX2 (Addgene plasmid #12260) and pMD2.G (Addgene plasmid #12259) in HEK293T cells, and viral particles were used to transduce AsPc-1, BxPc-3, Capan-1, and DanG cells, followed by puromycin selection. Generation of IRF1 CRISPR/Cas9 viral particles was reported previously [30]. KO efficiency was confirmed using western blot analysis.

### Statistical analysis

Statistical significance, when comparing two groups, was assessed by Student’s *t*-test (two-tailed distribution, two-sample, equal variance) using Microsoft Excel (Microsoft Deutschland GmbH, Unterschleissheim, Germany). Data are considered significant as *p < 0.05, **p < 0.01, ***p < 0.001.

## Results

### STING activation cooperates with BV6 to induce necroptosis in apoptosis-deficient PC cell lines

**Fig. 1:**
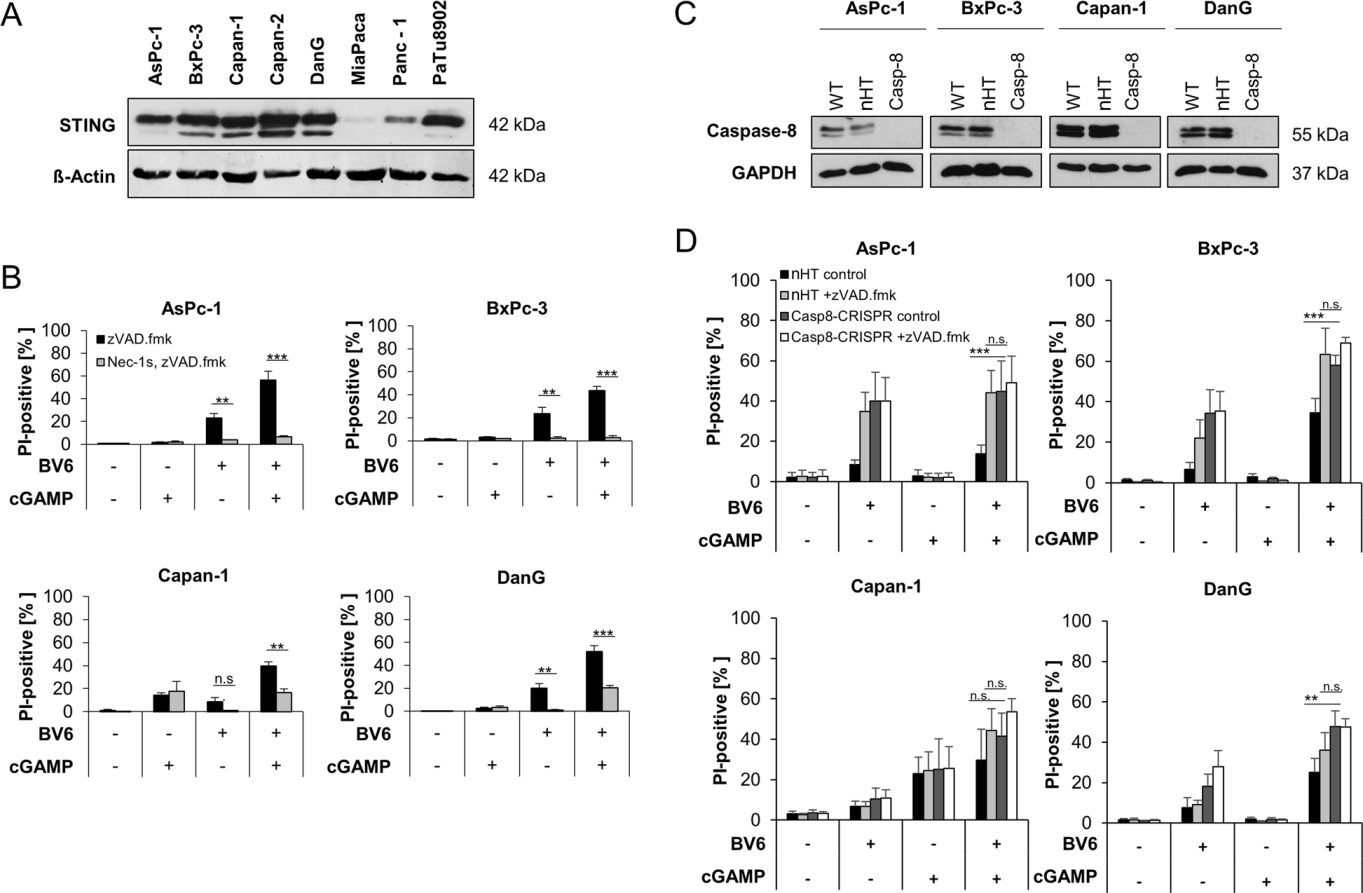
STING activation cooperates with BV6 to induce necroptosis in apoptosis-deficient PC cell lines. **A** Western blot analysis of STING expression in the indicated PC cell lines. β-Actin serves as loading control. Representative blots of at least two different independent experiments are shown. **B** AsPc-1, BxPc-3, Capan-1, and DanG cells were pretreated for 10 min with 2′3′-cGAMP (4 µg/ml) and/or treated with BV6 (5 µM) for 48 h in the presence of 20 μM zVAD.fmk with or without 20 μM Nec-1s. The amount of cell death was determined by quantifying propidium iodide (PI) uptake determined with the ImageXpress Micro XLS system. Data are presented as percentage of PI-positive cells, and mean and SD of three independent experiments performed in triplicate are shown. **P < 0.01, ***P < 0.001, n.s., not significant. **C** Western blot analysis of caspase-8 expression in the wild-type (WT), non-human target (nHT), and CRISPR/Cas9-mediated caspase-8 KO in the indicated PC cell lines. GAPDH serves as loading control. Representative blots of at least two different independent experiments are shown. **D** The indicated nHT and caspase-8 KO (Casp8-CRISPR) AsPc-1, BxPc-3, Capan-1, and DanG cells were pretreated for 10 min with 2′3′-cGAMP (4 µg/ml) and/or treated with BV6 (5 µM) for 48 h in the presence of 20 μM zVAD.fmk. The amount of cell death was determined by quantifying PI uptake determined with the ImageXpress Micro XLS system. Data are presented as percentage of PI-positive cells, and mean and SD of three independent experiments performed in triplicate are shown. **P < 0.01, ***P < 0.001; n.s., not significant.

To understand the role of STING signaling in PC cell lines, western blot analysis was performed to analyze STING expression in a selection of PC cell lines. From this panel, AsPc-1, BxPc-3, Capan-1, and DanG cells were used for further experiments, based on STING expression (Fig. 1A) and expression of RIPK3 and MLKL, which enable these cells to undergo necroptosis [5].

To investigate whether activation of STING signaling might prime apoptosis-deficient PC cells for necroptotic cell death, apoptosis resistance was mimicked by the use of the broad-range caspase inhibitor zVAD.fmk. In these settings, STING activation by 2′3′-cGAMP cooperated with BV6 to trigger cell death in PC cells (Fig. 1B). Importantly, 2′3′-cGAMP/BV6/zVAD.fmk-induced cell death exceeded the amount of cell death triggered by BV6 alone, whereas treatment with 2′3′-cGAMP alone exerted little cytotoxicity (Fig. 1B). Notably, 2′3-cGAMP/BV6/zVAD.fmk-induced cell death could be reduced by Nec-1s-mediated inhibition of RIPK1 in all PC cell lines tested (Fig. 1B), pointing towards RIPK1-dependent necroptotic cell death. Exposure of the PC cell lines to zVAD.fmk did not trigger cell death on its own as compared to untreated controls (Supplementary Fig. 1A, B). As zVAD.fmk-mediated inhibition of apoptosis only mimics apoptosis resistance partially and in a relatively artificial manner, we applied CRISPR/Cas9-mediated KO of caspase-8 in AsPc-1, BxPc-3, Capan-1, and DanG PC cell lines to mimic apoptosis resistance (Fig. 1C). Prominent 2′3′-cGAMP/BV6-induced cell death could be detected in caspase-8-deficient PC cell lines, confirming the effects observed with 2′3′-cGAMP/BV6/zVAD.fmk and excluding potential side effects of zVAD.fmk addition (Fig. 1D).

### BV6 and 2′3′-cGAMP induce immunostimulatory signaling in PC cells

**Fig. 2:**
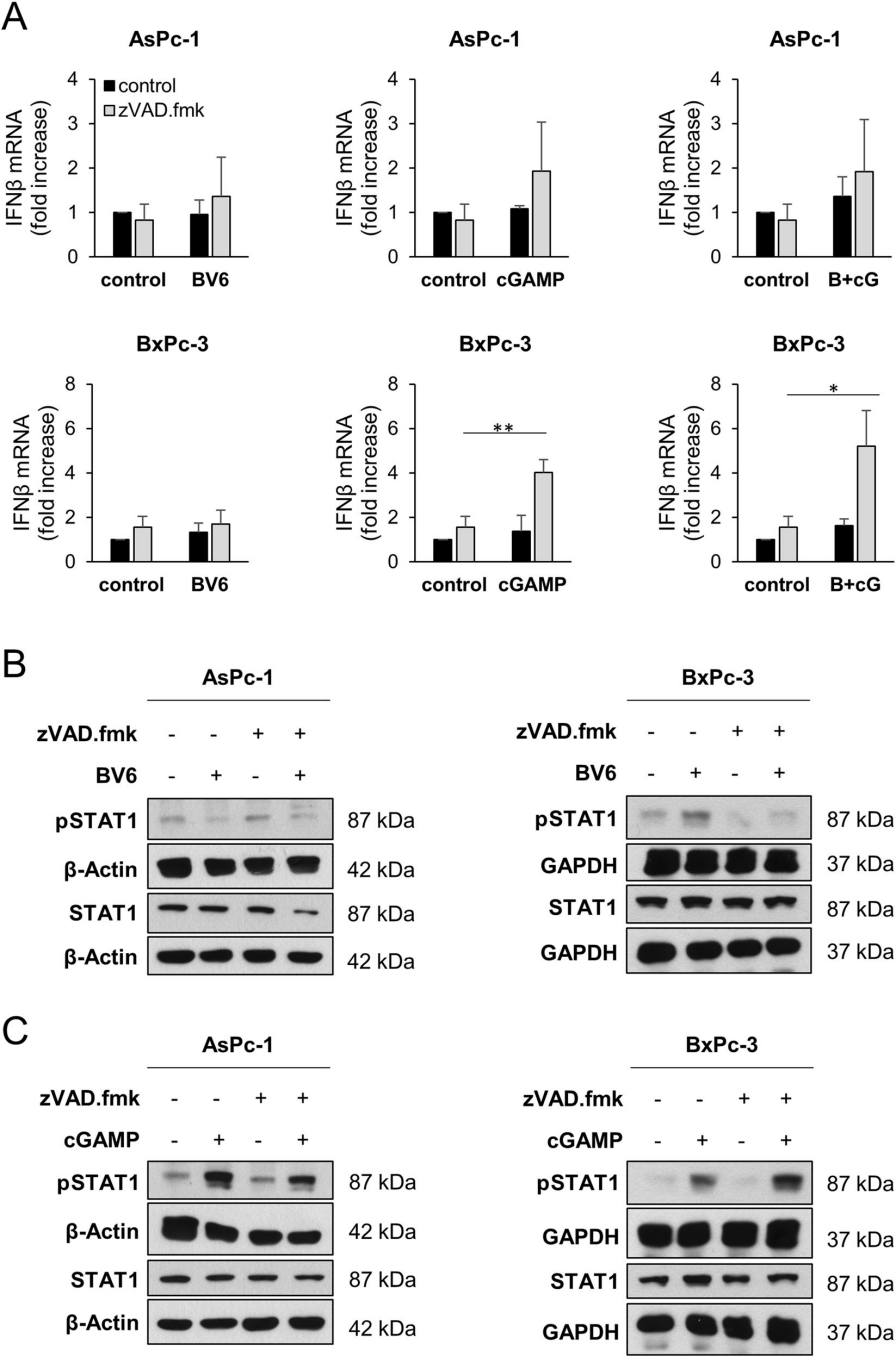
BV6 and 2′3′-cGAMP induce immunostimulatory signaling. **A** mRNA expression levels of IFNβ were determined in the indicated cell lines subjected to 4 µg/ml 2′3′-cGAMP or 5 µM BV6 for 8 h in the absence or presence of 20 μM zVAD.fmk using qRT-PCR. Data are normalized to GAPDH expression and are presented as x-fold mRNA expression compared to control. Mean and SD of three (BxPc-3) or four (AsPc-1) independent experiments performed in triplicate are shown. *P < 0.05, **P < 0.01. **B** Western blot analysis of phosphorylated STAT1 (pSTAT1) and total STAT1 in the indicated PC cell lines treated with 5 µM BV6 for 6 h in the absence or presence of 20 μM zVAD.fmk. GAPDH or β-actin served as loading controls. Representative blots of at least two different independent experiments are shown. **C** Western blot analysis of pSTAT1 and total STAT1 in the indicated PC cell lines treated with 4 µg/ml 2′3′-cGAMP for 6 h in the absence or presence of 20 μM zVAD.fmk. GAPDH or β-actin serves as loading controls. Representative blots of at least two different independent experiments are shown.

STING activation induces IFN production in tumor cells [19]. In addition, BV6 has been shown to exert immunomodulating activities, including the induction of host immune response [31, 32] and tumor cell-autonomous type I IFN responses [33, 34]. Therefore, we determined alterations in IFNβ mRNA expression levels in PC cells in response to 2′3′-cGAMP and BV6 alone or as a combination in the presence or absence of zVAD.fmk. In both AsPc-1 and BxPc-3 cells, increased IFNβ mRNA could be detected upon treatment with 2′3′-cGAMP or a combination of BV6/2′3′-cGAMP in the presence of zVAD.fmk, compared to the controls (Fig. 2A), whereas these effects were less obvious in the absence of zVAD.fmk or with BV6 alone (Supplementary Fig. 2A). Preventing necroptotic cell death by pharmacological inhibition of RIPK3 using GSK’872 slightly dampened BV6, 2′3′-cGAMP-, or BV6/2′3′-cGAMP-induced IFNβ mRNA levels (Supplementary Fig. 2B).

As IFNs signal through signal transducer and activator of transcription-1 (STAT1), we determined the phosphorylation status of STAT1. In line with our previous observations, phospho-STAT1 levels were shown to be elevated upon treatment with 2′3′-cGAMP/zVAD.fmk but not with BV6/zVAD.fmk, compared to the controls in AsPc-1 and BxPc-3 (Fig. 2B, C and Supplementary Fig. 3A). Similarly, phosphorylated STAT1 levels were increased in 2′3′-cGAMP-treated caspase-8 KO AsPc-1 and BxPc-3 cells, but not in BV6-treated cells, excluding unanticipated effects of zVAD.fmk (Supplementary Fig. 3B). These experiments suggest immunostimulatory effects of the STING agonist 2′3′-cGAMP cells in apoptosis-deficient PC cell lines.

### BV6 synergizes with type I and type II IFNs in apoptosis-resistant PC cells to induce necroptotic cell death

**Fig. 3:**
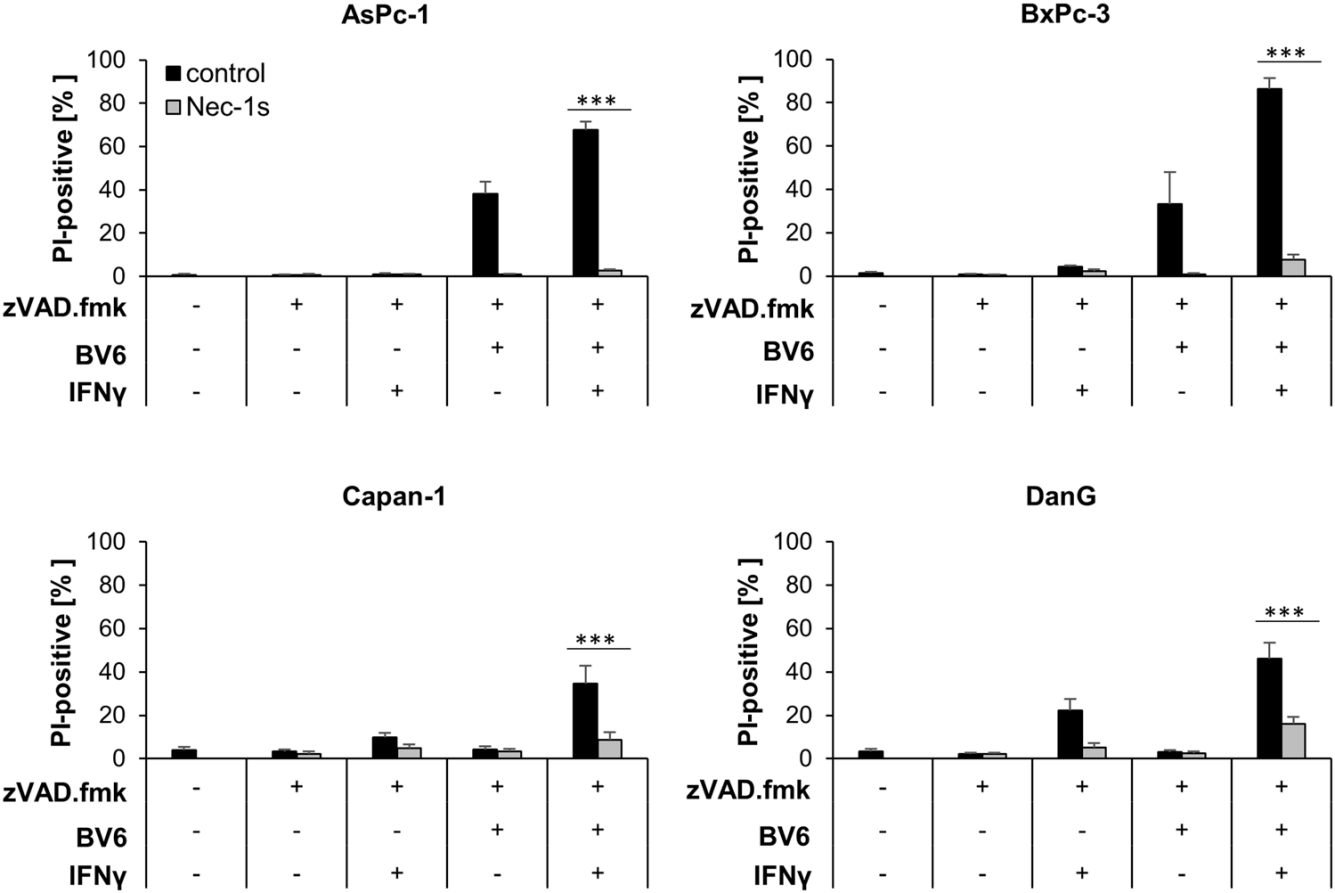
BV6 synergizes with IFNγ to induce caspase-independent cell death in apoptosis-deficient PC cells. AsPc-1, BxPc-3, Capan-1, and DanG cells were treated with 5 µM BV6 and/or 3 ng/ml IFNγ for 48 h in the presence of 20 μM zVAD.fmk with or without 20 μM Nec-1s. The amount of cell death was calculated by quantifying PI uptake determined with the ImageXpress Micro XLS system. Data are presented as percentage of PI-positive cells, and mean and SD of three independent experiments performed in triplicate are shown. ***P < 0.001.

Next, we investigated whether IFNs are able to prime PC cells for BV6-triggered cell death. To this end, PC cells were exposed to BV6 and/or type I or II IFNs upon caspase inhibition, followed by quantification of cell death. Importantly, BV6 acted in concert with IFNγ and 2′3′-cGAMP (Fig. 3 and Supplementary Fig. 4A), as well as IFNβ (Supplementary Fig. 4B), to induce cell death in the presence of zVAD.fmk in AsPc-1 and BxPc-3 PC cell lines. This increase in cell death triggered by IFN/BV6/zVAD.fmk could be efficiently suppressed by Nec-1s, suggesting necroptotic cell death (Fig. 3 and Supplementary Fig. 4B).

**Fig. 4:**
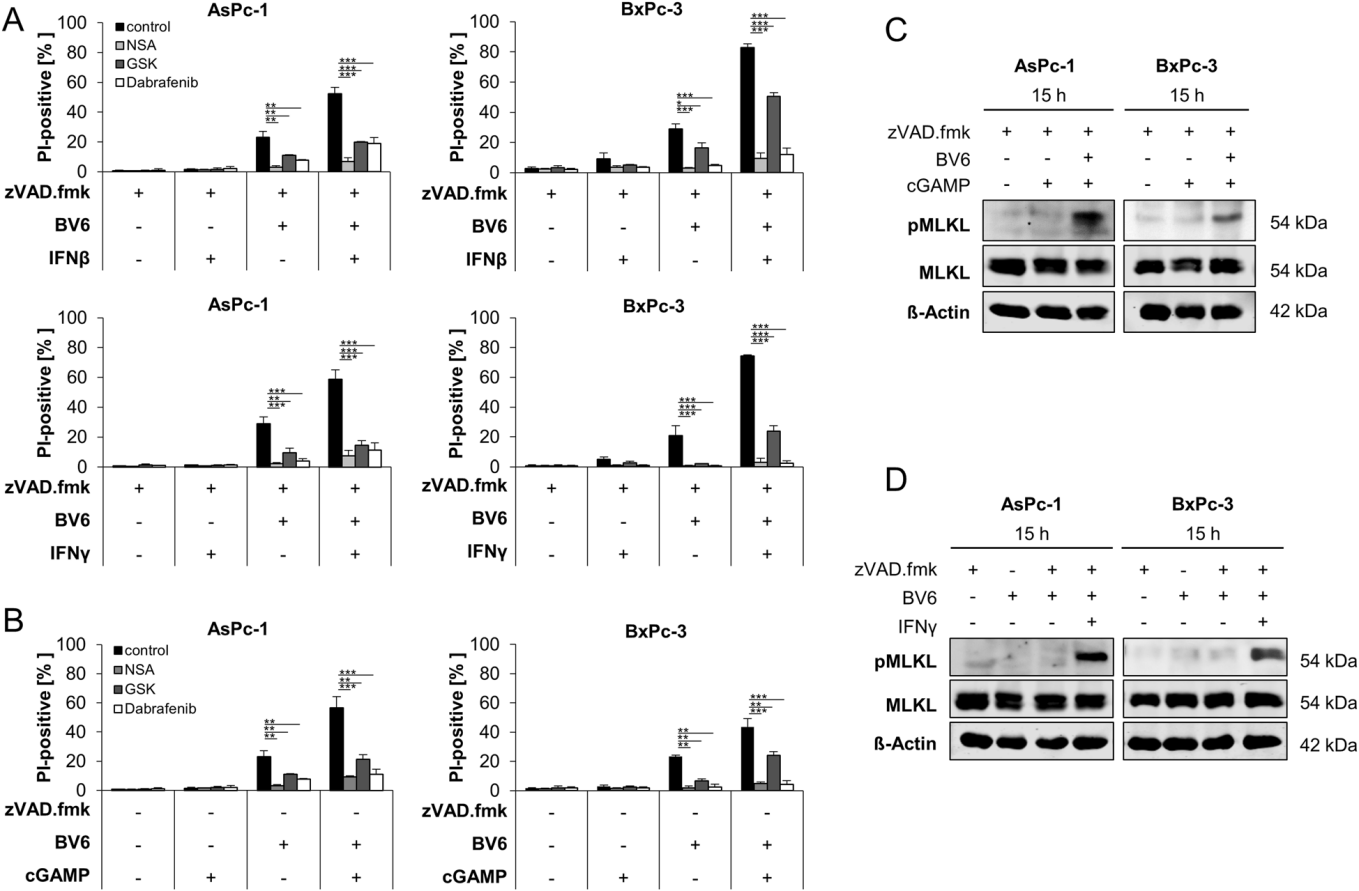
Co-treatment with BV6 and 2′3′-cGAMP or IFNs induces necroptotic cell death upon caspase inhibition. **A** AsPc-1 and BxPc-3 cells were treated with 20 μM zVAD.fmk, 5 µM BV6, 10 ng/ml IFNβ, or 3 ng/ml IFNγ for 48 h in the presence or absence of 1 µM NSA, 5 µM Dabrafenib, or 10 µM GSK’872. The amount of cell death was calculated by quantifying PI uptake determined with the ImageXpress Micro XLS system. Data are presented as percentage of PI-positive cells, and mean and SD of three independent experiments performed in triplicate are shown. *P < 0.05; **P < 0.01, ***P < 0.001, n.s., not significant. **B** AsPc-1 and BxPc-3 cells were treated with 20 μM zVAD.fmk, 5 µM BV6, and/or 4 µg/ml 2′3′-cGAMP for 48 h in the presence or absence of 1 µM NSA, 5 µM Dabrafenib, or 10 µM GSK’872. The amount of cell death was calculated by quantifying PI uptake determined with the ImageXpress Micro XLS system. Data are presented as percentage of PI-positive cells and mean and SD of three independent experiments performed in triplicate are shown. **P < 0.01, ***P < 0.001. **C** Western blot analysis of phosphorylated MLKL (pMLKL) and total MLKL in the indicated PC cell lines treated with 4 µg/ml 2′3′-cGAMP and/or 5 µM BV6 for 15 h in the presence of 20 μM zVAD.fmk. β-Actin served as loading control. Representative blots of at least two different independent experiments are shown. **D** Western blot analysis of pMLKL and total MLKL in the indicated PC cell lines treated with 3 ng/ml IFNγ and/or 5 µM BV6 in the absence or presence of 20 μM zVAD.fmk. β-Actin served as loading control. Representative blots of at least two different independent experiments are shown.

To confirm that IFN/BV6/zVAD.fmk co-treatment does indeed trigger necroptosis, the relevance of RIPK3 and MLKL, crucial mediators of necroptosis, were assessed. Pharmacological inhibition of RIPK3 activity using GSK’872 or Dabrafenib impeded IFNβ/BV6/zVAD.fmk- and IFNγ/BV6/zVAD.fmk-mediated cell death in AsPc-1 and BxPc-3 PC cell lines (Fig. 4A). Similarly, the MLKL inhibitor NSA also reduced IFNβ/BV6/zVAD.fmk- and IFNγ/BV6/zVAD.fmk-induced cell death (Fig. 4A). In addition, 2′3′-cGAMP/BV6/zVAD.fmk-mediated cell death was also decreased by pharmacological inhibition of RIPK3 and MLKL in all tested PC cell lines (Fig. 4B). As expected, phosphorylation of MLKL, a key characteristic of necroptosis [7], could be detected after treatment with 2′3′-cGAMP/BV6/zVAD.fmk or IFNγ/BV6/zVAD.fmk already after 15 h (Fig. 4C, D and Supplementary Fig. 5). These findings confirm that BV6 combined with IFNs or 2′3′-cGAMP trigger necroptotic cell death in apoptosis-deficient PC cells.

### STING is required for 2′3′-cGAMP/BV6/zVAD.fmk-induced cell death

**Fig. 5:**
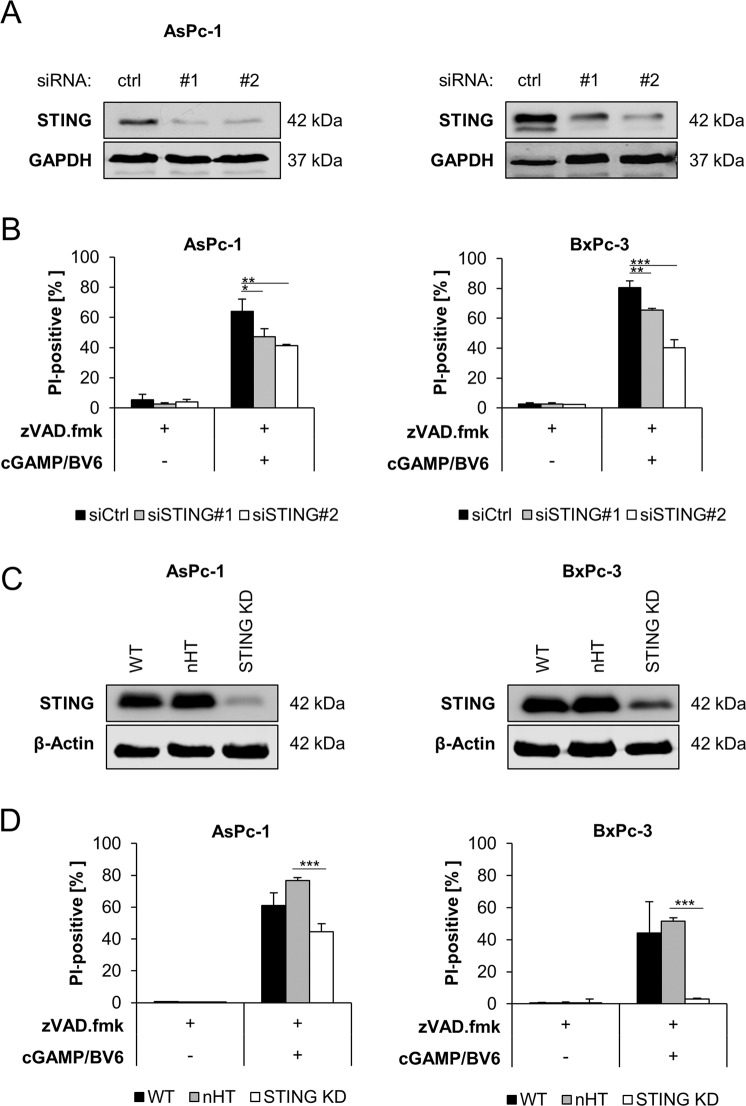
STING is required for 2′3′-cGAMP/BV6/zVAD.fmk-induced cell death. **A** Western blot analysis of STING expression in the indicated PC cell lines after 48 h of transfection with non-silencing RNA (ctrl) or siRNA targeting STING. GAPDH serves as loading control. Representative blots of at least two different independent experiments are shown. **B** AsPc-1 and BxPc-3 cells were transfected with control siRNA (siCtrl) or two independent siRNAs targeting STING and were treated with 20 μM zVAD.fmk for 48 h in the presence or absence of 4 µg/ml 2′3′-cGAMP and 5 µM BV6. The amount of cell death was calculated by quantifying PI uptake determined with the ImageXpress Micro XLS system. Data are presented as percentage of PI-positive cells, and mean and SD of three independent experiments performed in triplicate are shown. **P < 0.01, ***P < 0.001. **C** Western blot analysis of AsPc-1 and BxPc-3 subjected to nHT and STING CRISPR/Cas9 knockdown. β-Actin serves as loading control. Representative blots of at least two different independent experiments are shown. **D** AsPc-1 and BxPc-3 cells were subjected to nHT and STING CRISPR/Cas9 knockdown, and treated with 20 μM zVAD.fmk for 48 h in the presence or absence of 4 µg/ml 2′3′-cGAMP and 5 µM BV6. The amount of cell death was calculated by quantifying PI uptake determined with the ImageXpress Micro XLS system. Data are presented as percentage of PI-positive cells, and mean and SD of three independent experiments performed in triplicate are shown. ***P < 0.001.

To elucidate the signaling pathways that mediate necroptotic cell death upon 2′3′-cGAMP/BV6 co-treatment, we evaluated the requirement of STING/IFN signaling. First, we silenced STING using two distinct siRNA sequences, which resulted in downregulation of STING protein levels (Fig. 5A). Importantly, knockdown of STING considerably reduced 2′3′-cGAMP/BV6/zVAD.fmk-stimulated cell death (Fig. 5B). Besides transient knockdown of STING by siRNA, we also applied CRISPR/Cas9-mediated reduction of STING expression, leading to a substantial decrease in STING expression (Fig. 5C) and resulted in a significant decrease of cell death upon treatment with 2′3′-cGAMP/BV6/zVAD.fmk compared to the nHT control (Fig. 5D). Of note, transfection with STING siRNA consistently rendered BxPc-3 cells more sensitive to treatment with BV6/zVAD.fmk, compared to STING CRISPR/Cas9. Together, these findings demonstrate that STING is required for 2′3′-cGAMP/BV6/zVAD.fmk-induced cell death.

### NF-κB signaling contributes to 2′3′-cGAMP/BV6/zVAD.fmk-induced cell death

**Fig. 6:**
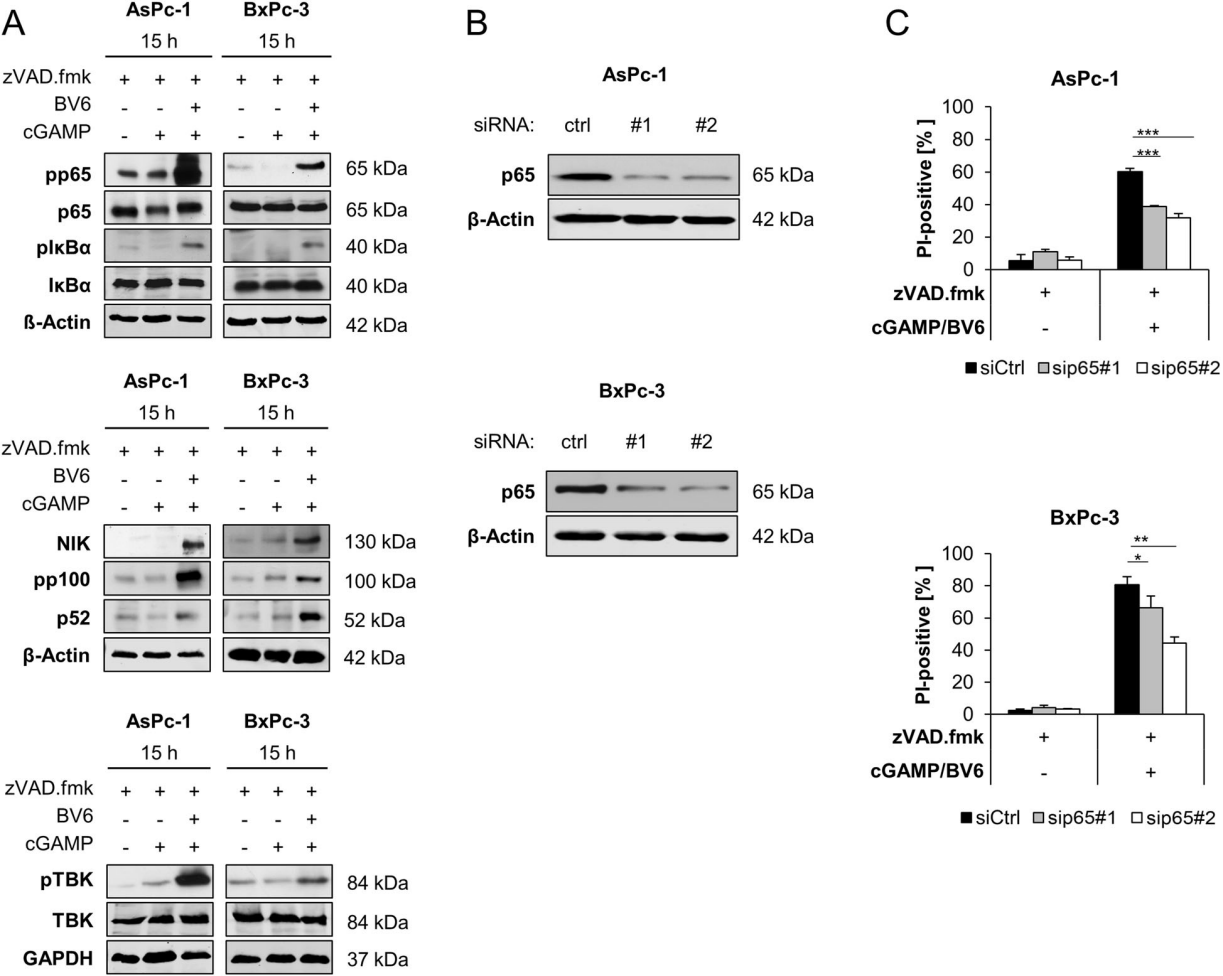
NF-κB signaling contributes to 2′3′-cGAMP/BV6/zVAD.fmk-induced cell death. **A** Western blot analysis of phosphorylated and total p65, IκBα, NIK, p100, p52, and TBK1 in the indicated cell lines treated with 4 µg/ml 2′3′-cGAMP and/or 5 µM BV6 for 15 h in the presence or absence of 20 μM zVAD.fmk. GAPDH or β-Actin serves as loading controls. Representative blots of at least two different independent experiments are shown. **B** Western blot analysis of p65 in the indicated PC cell lines after 48 h of transfection with non-silencing RNA (ctrl) or siRNA targeting p65. β-Actin serves as loading control. Representative Western blots of at least two different independent experiments are shown. **C** AsPc-1 and BxPc-3 cells were transfected with control siRNA (siCtrl) or two independent siRNAs targeting p65 and were treated with 4 µg/ml 2′3′-cGAMP and 5 µM BV6 for 48 h in the presence of 20 μM zVAD.fmk. The amount of cell death was calculated by quantifying PI uptake determined with the ImageXpress Micro XLS system. Data are presented as percentage of PI-positive cells, and mean and SD of three independent experiments performed in triplicate are shown. *P < 0.05, **P < 0.01, ***P < 0.001.

Next, we investigated the involvement of NF-κB signaling upon treatment with 2′3′-cGAMP/BV6/zVAD.fmk and BV6/zVAD.fmk. Exposure of AsPc-1 and BxPc-3 cells to 2′3′-cGAMP/BV6/zVAD.fmk and BV6/zVAD.fmk induced phosphorylation of p65, IκBα, p100, and TBK1, as well as accumulation of NIK, indicating an activation of canonical and non-canonical NF-κB signaling pathways (Fig. 6A and Supplementary Fig. 6). STING activation via 2′3′-cGAMP does not lead to major activation of NF-κB signaling (Fig. 6A). For further investigation of the functional role of NF-κB in 2′3′-cGAMP/BV6/zVAD.fmk-induced cell death, we silenced p65 using two distinct siRNA sequences, resulting in downregulation of p65 protein in both AsPc-1 and BxPc-3 cell lines (Fig. 6B). Of note, p65 knockdown significantly rescued 2′3′-cGAMP/BV6/zVAD.fmk-mediated cell death in both cell lines (Fig. 6C), suggesting that NF-κB signaling is involved in 2′3′-cGAMP/BV6/zVAD.fmk-triggered cell death.

### The TNFα autocrine/paracrine loop is involved in cGAMP/BV6-induced cell death

**Fig. 7:**
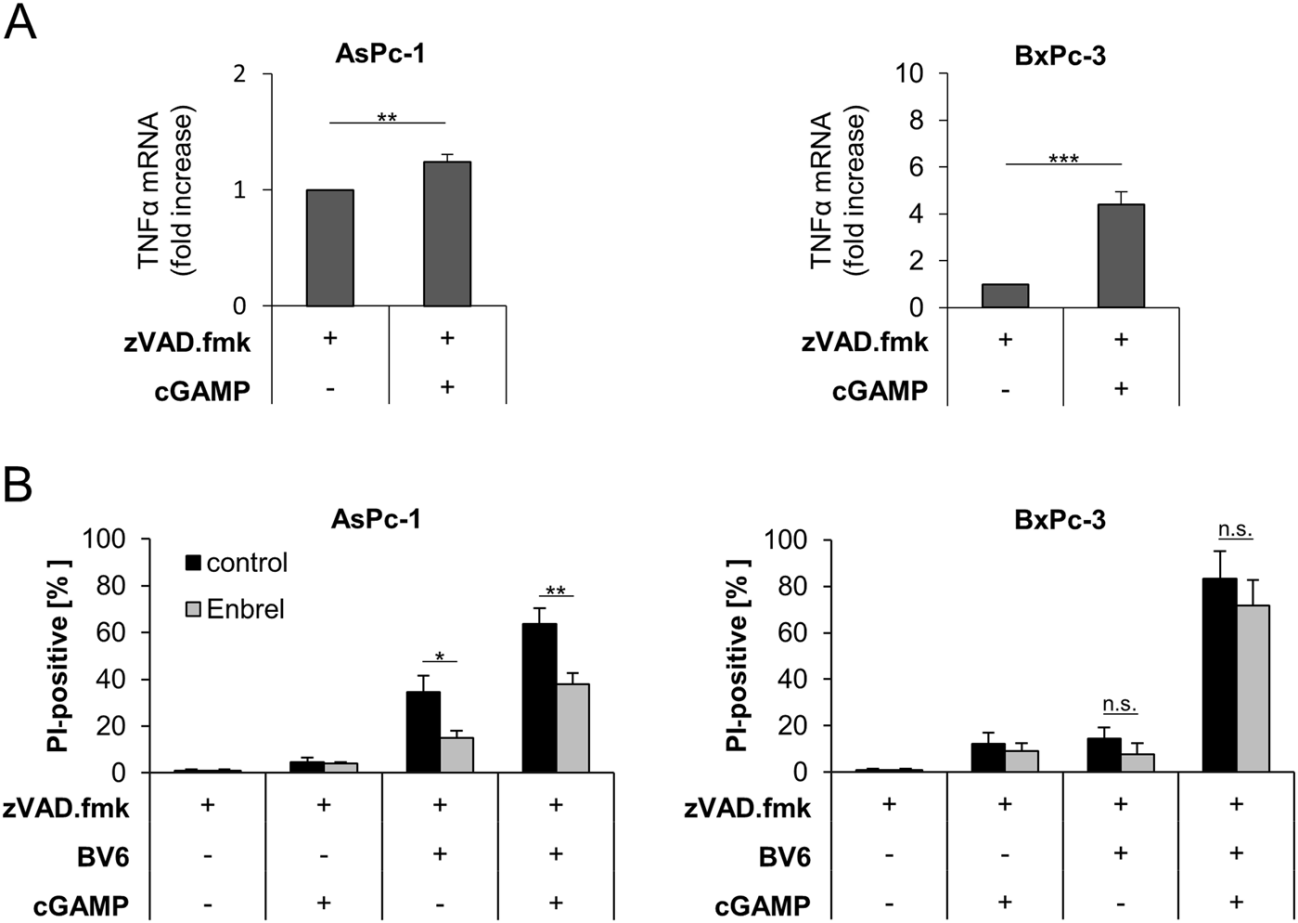
The TNFα autocrine/paracrine loop is involved in cGAMP/BV6-induced cell death. **A** mRNA expression levels of TNFα were determined in the indicated cell lines subjected to 4 µg/ml 2′3′-cGAMP for 10 min in the presence of 20 μM zVAD.fmk using qRT-PCR. Data are normalized to GAPDH expression and are presented as x-fold mRNA expression compared to control. Mean and SD of three independent experiments performed in triplicate are shown. **P < 0.01, ***P < 0.001. **B** AsPc-1 and BxPc-3 cells were treated with 4 µg/ml 2′3′-cGAMP for 10 min and/or 5 µM BV6 for 48 h in the presence of 20 μM zVAD.fmk and in the absence or presence of 50 µg/ml Enbrel. The amount of cell death was calculated by quantifying PI uptake determined with the ImageXpress Micro XLS system. Data are presented as percentage of PI-positive cells, and mean and SD of three independent experiments performed in triplicate are shown. *P < 0.05; **P < 0.01, n.s., not significant.

Previously, we have demonstrated that, as a result of NF-κB activation by BV6, paracrine/autocrine TNFα is a central driving force of BV6-induced necroptosis in apoptosis-deficient PC cells [5]. Therefore, we tested the hypothesis that 2′3′-cGAMP/BV6/zVAD.fmk-induced necroptosis also depends on TNFα signaling. To address this point, we first determined TNFα mRNA levels upon treatment with 2′3′-cGAMP, and both AsPc-1 and BxPc-3 cells displayed increased TNFα mRNA expression levels, although to a variable extent (Fig. 7A). Interestingly, antagonizing TNFα with Enbrel diminished both BV6/zVAD.fmk- and 2′3′-cGAMP/BV6/zVAD.fmk-mediated cell death in AsPc-1 cells and to a lesser extent in BxPc-3 cells (Fig. 7B). These findings suggest that 2′3′-cGAMP/BV6/zVAD.fmk-induced necroptosis is potentiated by autocrine/paracrine TNFα signaling.

### IRF1 contributes to 2′3′-cGAMP/BV6/zVAD.fmk-induced cell death

**Fig. 8:**
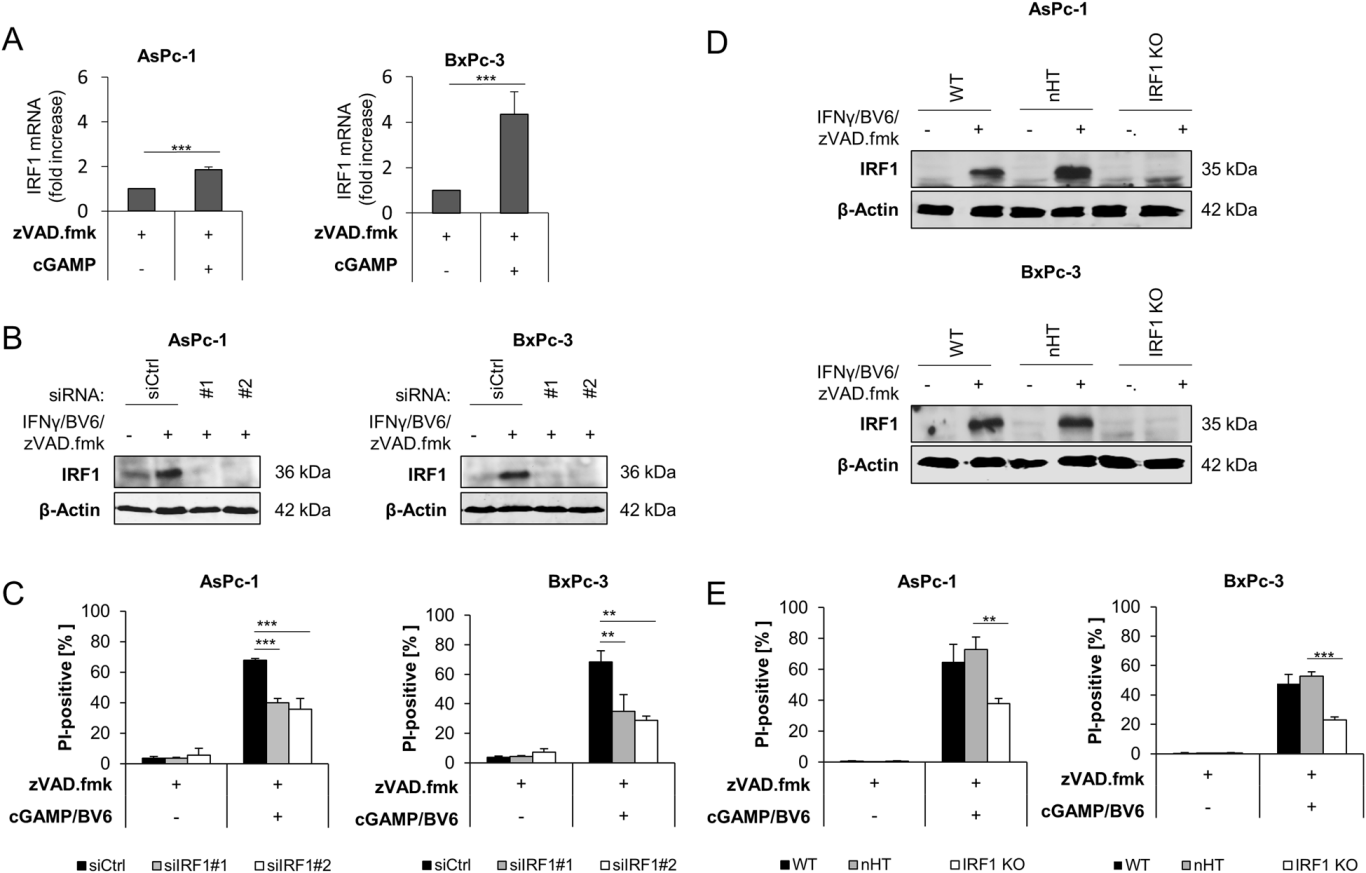
IRF1 contributes to 2′3′-cGAMP/BV6/zVAD.fmk-induced cell death. **A** mRNA expression levels of IRF1 were determined in the indicated cell lines subjected to 4 µg/ml 2′3′-cGAMP in the presence of 20 μM zVAD.fmk using qRT-PCR. Data are normalized to GAPDH expression and are presented as *x*-fold mRNA expression compared to control. Mean and SD of three independent experiments performed in triplicate are shown. ***P < 0.001. **B** Western blot analysis of IRF1 in indicated PC cell lines after 48 h of transfection with non-silencing RNA (siCtrl) or siRNA targeting IRF1 that were left treated with 5 µM BV6, 3 ng/ml IFNγ, and 20 µM zVAD.fmk for 15 h. β-Actin serves as loading control. Representative blots of at least two different independent experiments are shown. **C** AsPc-1 and BxPc-3 cells were transfected with control siRNA (siCtrl) or two independent siRNAs targeting IRF1 and were treated with 4 µg/ml 2′3′-cGAMP and 5 µM BV6 for 48 h in the presence of 20 μM zVAD.fmk. The amount of cell death was calculated by quantifying PI uptake determined with the ImageXpress Micro XLS system. Data are presented as percentage of PI-positive cells, and mean and SD of three independent experiments performed in triplicate are shown. **P < 0.01, ***P < 0.001. **D** Western blot analysis of nHT and IRF1 CRISPR/Cas9 KO in indicated PC cell lines that were treated with 5 µM BV6, 3 ng/ml IFNγ, and 20 µM zVAD.fmk for 15 h. β-Actin serves as loading control. Representative blots of at least two different independent experiments are shown. **E** AsPc-1 and BxPc-3 cells were subjected to nHT and IRF1 CRISPR/Cas9 knockdown, and treated with 4 µg/ml 2′3′-cGAMP and/or 5 µM BV6 for 48 h in the presence of 20 μM zVAD.fmk. The amount of cell death was calculated by quantifying PI uptake determined with the ImageXpress Micro XLS system. Data are presented as percentage of PI-positive cells and mean and SD of three independent experiments performed in triplicate are shown. **P < 0.01, ***P < 0.001.

Finally, we investigated the role of IRF1, a central transcription factor involved in IFN responses that has been implicated in necroptosis in a variety of cancer cells [15, 30]. During IFNγ/BV6/zVAD.fmk-mediated necroptosis, IRF1 mRNA levels (Fig. 8A) and protein levels (Supplementary Fig. 7) were increased. To further explore the role of IRF1 in 2′3′-cGAMP/BV6/zVAD.fmk-induced cell death, we transiently silenced IRF1 by siRNA (Fig. 8B). Importantly, knockdown of IRF1 resulted in a significant reduction of 2′3′-cGAMP/BV6/zVAD.fmk-triggered cell death compared to non-silencing controls in both cell lines (Fig. 8C). Likewise, IRF1 KO cells generated by CRISPR/Cas9-mediated editing also demonstrated a significant decrease in cell death induced by 2′3′-cGAMP/BV6/zVAD.fmk compared to controls (Fig. 8D, E). Taken together, these findings show that IRF1 contributes to 2′3′-cGAMP/BV6/zVAD.fmk-mediated cell death.

## Discussion

Currently, surgery remains the only curative option for patients suffering from PC, but only a minority of cases are eligible for tumor resection [35]. Therefore, there is a high demand for developing novel, effective systemic therapies against PC to improve the dismal survival of only a few months in those patients.

In the present study, we demonstrate that the DNA sensor STING can be engaged in PC cells to induce the expression of IFN responses. STING activation via the exogenous STING agonist 2′3′-cGAMP and treatment with IFNs induced necroptotic cell death in apoptosis-resistant PC cells when combined with the Smac mimetic BV6. Smac mimetics as single agents are generally ineffective in most tumors [36]. However, the present study provides evidence showing that a combination therapy with BV6 and STING agonists might be a promising future strategy against unresponsive PC. Exploiting those compounds in a dual immunostimulatory approach might be a potent strategy to overcome treatment resistance in “non-immunogenic” tumors such as PC. Activation of IFN signaling and expression of IFN-stimulated genes have been shown to contribute to improve prognosis in cancer [37]. PC is generally considered a “non-immunogenic” type of tumor and a strong desmoplastic reaction impedes treatment response. Hence, recent immunotherapeutic approaches have not been successful in PC [38]. Recently, it has been suggested that exploiting STING activation might be an effective anticancer strategy in PC to stimulate the immunologically suppressed microenvironment [39–41]. STING agonists enhanced treatment response of pancreatic tumors, which were insensitive to radiation [39] or standard-of-care chemotherapy [40] by inducing host immunosurveillance. STING agonist monotherapy activated host anticancer immunity responses leading to a regression of pancreatic tumors in a transgenic mouse model of PC [40]. In addition, STING activation by the cancer vaccine STINGVAX triggered antitumor responses in various cancer types, including PC, and resulted in susceptibility to immunotherapy in weakly immunogenic tumors [41]. Our study confirms the significance of STING activation in PC cells and its role in inducing upregulation of IFNβ, a signature cytokine of the cGAS/STING pathway [17, 18]. Notably, our findings suggest that STING activation sensitizes apoptosis-resistant PC cells for necroptosis. We confirmed the mode of cell death by pharmacological inhibition of necroptotic mediators (RIPK1, RIPK3, MLKL) and by monitoring MLKL phosphorylation, a key event in the execution of necroptosis. Likewise, in bone marrow-derived macrophages, necroptosis occurred downstream of STING activation, triggered by synergistic type I IFN and TNFα signaling, and mediated by NF-κB [42]. Our study highlights the relevance of NF-κB and TNFα as mediators of 2′3′-cGAMP/BV6/zVAD.fmk-induced necroptosis; however, agonizing STING with 2′3′-cGAMP did not induce NF-κB signaling. In addition, we could demonstrate that IRF1 contributes to 2′3′-cGAMP/BV6/zVAD.fmk-mediated necroptosis in PC. IRF1 plays a crucial role in type I IFNs signaling and immunity as revealed by the involvement of IRF1 in various diseases, including cancer [37, 43]. Activation of the IFN signaling cascade and IFN target genes play important roles in human malignancies [37]. In PC, IRF1 has been identified as an anti-oncoprotein and its overexpression has been associated with better clinic-pathological features, such as tumor differentiation, infiltration depth, tumor size, and survival time [44].

Interestingly, type I and II IFNs are known to upregulate MLKL in an IRF1-dependent manner, suggesting that upregulation of IRF1 might affect necroptosis [30]. Consistently, it has been demonstrated in previous studies that IRF1 plays an important role in triggering necroptosis induced by IFNγ and Smac mimetics upon inhibition of caspases [15]. In addition, IRF1 contributes to BV6-mediated cell death and attenuates its pro-inflammatory response by controlling the induction of NF-κB target genes [44, 45].

In summary, this study suggests that future dual immunostimulatory approaches, combining Smac mimetics with STING activation, might be a powerful anticancer strategy to overcome treatment failure in PC. The efficacy of this combination regimen has to be carefully examined, as the inflammatory response is unavoidably affected by immune cells in the tumor microenvironment [17, 44], as well as secretion of pro-inflammatory cytokines, which might affect treatment responses [36, 42, 46].

## Supplementary information

Supplemental Figure Legends

Supplemental Figure 1: Induction of cell death by BV6, cGAMP and IFNβ in the absence or presence of zVAD.fmk in PC cell lines

Supplemental Figure 2: BV6 and 2′3′-cGAMP trigger IFN induction in PC cell lines

Supplemental Figure 3: 2′3′-cGAMP and BV6-induce STAT1 phosphorylation in apoptosis-deficient PC cell lines

Supplemental Figure 4: Time course analysis of IFN-, 2′3′-cGAMP- and BV6-induced cell death

Supplemental Figure 5: Time course analysis of IFNγ-, 2′3′-cGAMP- and BV6-induced MLKL phosphorylation

Supplemental Figure 6: BV6 induces NF-κB signaling in PC cell lines

Supplemental Figure 7: BV6/IFNγ/zVAD.fmk-mediated upregulation of IRF1

Supplemental Tables

## Data Availability

Primary data are available on request from the corresponding author.
