# Supplementary material for: The Smac mimetic BV6 cooperates with STING to induce necroptosis in apoptosis-resistant pancreatic carcinoma cells
Source: Cell Death Dis. 2021 Aug 30;12(9):816. doi: 10.1038/s41419-021-04014-x (PMC8405653; doi:10.1038/s41419-021-04014-x)
Supplement: Supplementary file 1 — Supplemental Figure Legends [file 41419_2021_4014_MOESM1_ESM.docx]

# Supplemental figure legends

**Supplemental Figure 1: Induction of cell death by BV6, cGAMP and IFNβ in the absence or presence of zVAD.fmk in PC cell lines**

**A.** AsPc-1, BxPc-3, Capan-1 and DanG cells were treated with 4 µg/ml 2'3'-cGAMP, 5 µM BV6, 20 μM zVAD.fmk with or without 20 μM Nec‑1s for 48 hours. The amount of cell death was calculated by quantifying PI uptake determined with the ImageXpress Micro XLS system. Data are presented as percentage of PI-positive cells and mean and SD of three independent experiments performed in triplicate are shown. ** P < 0.01, *** P < 0.001, n.s.: not significant.

**B.** AsPc-1, BxPc-3, Capan-1 and DanG cells were treated with 3 ng/ml IFNβ, 5 µM BV6, 20 μM zVAD.fmk with or without 20 μM Nec‑1s for 48 hours. The amount of cell death was calculated by quantifying PI uptake determined with the ImageXpress Micro XLS system. Data are presented as percentage of PI-positive cells and mean and SD of three independent experiments performed in triplicate are shown. * P < 0.05, ** P < 0.01, *** P < 0.001.

**Supplemental Figure 2: BV6 and 2'3'-cGAMP trigger IFN induction in PC cell lines**

**A.** mRNA expression levels of IFNβ were determined in BxPc-3 after treatment with 4 µg/ml 2'3'-cGAMP (*left*) or 5 µM BV6(*right*) for the indicated time points. Data are normalized to 28S expression and are presented as x-fold mRNA expression compared to control. Mean and SD of three independent experiments performed in triplicate are shown.

**B.** mRNA expression levels of IFNβ were determined in AsPc-1 and BxPc-3 cells after treatment with 5 µM BV6 (left), 4 µg/ml 2'3'-cGAMP (*middle)* or in combination *(right)* in the presence of 10 µM GSK’872 in the presence or absence of 20 μM zVAD.fmk for 8 hours. Data are normalized to 28S expression and are presented as x-fold mRNA expression compared to control. Mean and SD of three (BxPc-3) or four (AsPc-1) independent experiments performed in triplicate are shown.

**Supplemental Figure 3: 2'3'-cGAMP and BV6-induce STAT1 phosphorylation in apoptosis-deficient PC cell lines**

**A.** Western blot analysis of phosphorylated STAT1 (pSTAT1) and total STAT1 in the indicated PC cell lines treated with 20 µM zVAD.fmk alone or in combination with 4 µg/ml 2'3'-cGAMP or 5 µM BV6 for the indicated time points. Vinculin and β-Actin served as loading controls. Representative blots of at least two different independent experiments are shown.

**B.** Western blot analysis of phosphorylated STAT1 (pSTAT1) and total STAT1 in the indicated nHT and caspase-8 CRISPR/Cas9 KO PC cell lines treated with 4 µg/ml 2'3'-cGAMP or 5 µM BV6 for 6 hours in the absence or presence of 20 μM zVAD.fmk for the indicated time points. GAPDH served as loading control. Representative blots of at least two different independent experiments are shown.

**Supplemental Figure 4: Time course analysis of IFN-, 2'3'-cGAMP- and BV6-induced cell death**

**A.** AsPc-1, BxPc-3, Capan-1 and DanG cells were treated with 3 ng/ml IFNγ, 5 µM BV6 or 4 µg/ml 2'3'-cGAMP in the presence or absence of 20 μM zVAD.fmk for the indicated time points. The amount of cell death was calculated by quantifying PI uptake determined with the ImageXpress Micro XLS system. Data are presented as percentage of PI-positive cells and mean and SD of three independent experiments performed in triplicate are shown. * P < 0.05; ** P < 0.01, *** P < 0.001.

**B.** AsPc-1, BxPc-3, Capan-1 and DanG cells were treated with 3 ng/ml IFNβ and/or 5 µM BV6 in the presence of 20 μM zVAD.fmk with or without 20 μM Nec‑1s for 48 hours. The amount of cell death was calculated by quantifying PI uptake determined with the ImageXpress Micro XLS system. Data are presented as percentage of PI-positive cells and mean and SD of three independent experiments performed in triplicate are shown. * P < 0.05; ** P < 0.01, *** P < 0.001, n.s.: not significant.

**Supplemental Figure 5: Time course analysis of IFNγ-, 2'3'-cGAMP- and BV6-induced MLKL phosphorylation**

Western blot analysis of phosphorylated MLKL (pMLKL) and total MLKL in the indicated PC cell lines treated with zVAD.fmk alone or in combination with 4 µg/ml 2'3'-cGAMP, 3 ng/ml IFNγ or 5 µM BV6 for the indicated time-points. GAPDH served as loading control. Representative blots of at least two different independent experiments are shown.

**Supplemental Figure 6: BV6 induces NF-κB signaling in PC cell lines**

Western blot analysis of phosphorylated and total p65, NIK, p100, p52, IκBα and TBK1 in the indicated PC cell lines treated with zVAD.fmk and/or 5 µM BV6 for the indicated time points. GAPDH and β-Actin served as loading controls. Representative blots of at least two different independent experiments are shown.

**Supplemental Figure 7:** BV6/IFNγ/zVAD.fmk-mediated upregulation of IRF1

Western blot analysis of IRF1 in the indicated PC cell lines treated with zVAD.fmk and/or 5 µM BV6 with or without 3 ng/ml IFNγ for the indicated time points. GAPDH served as loading control. Representative blots of at least two different independent experiments are shown.
