# Supplementary figures and images for: The Smac mimetic BV6 cooperates with STING to induce necroptosis in apoptosis-resistant pancreatic carcinoma cells
Source: Cell Death Dis. 2021 Aug 30;12(9):816. doi: 10.1038/s41419-021-04014-x (PMC8405653; doi:10.1038/s41419-021-04014-x)

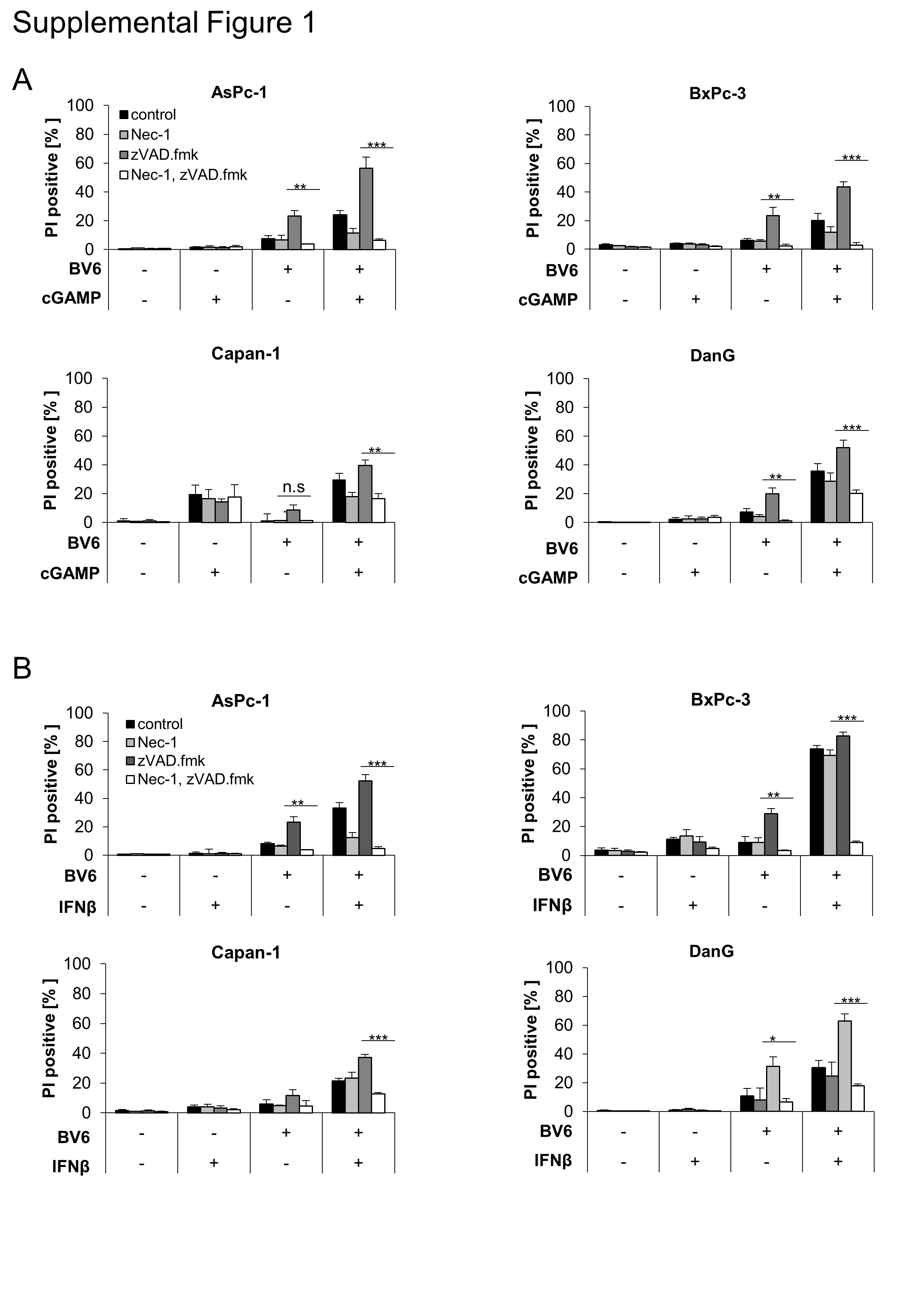

Supplement: Supplementary file 2 — Supplemental Figure 1: Induction of cell death by BV6, cGAMP and IFNβ in the absence or presence of zVAD.fmk in PC cell lines [file 41419_2021_4014_MOESM2_ESM.tif]

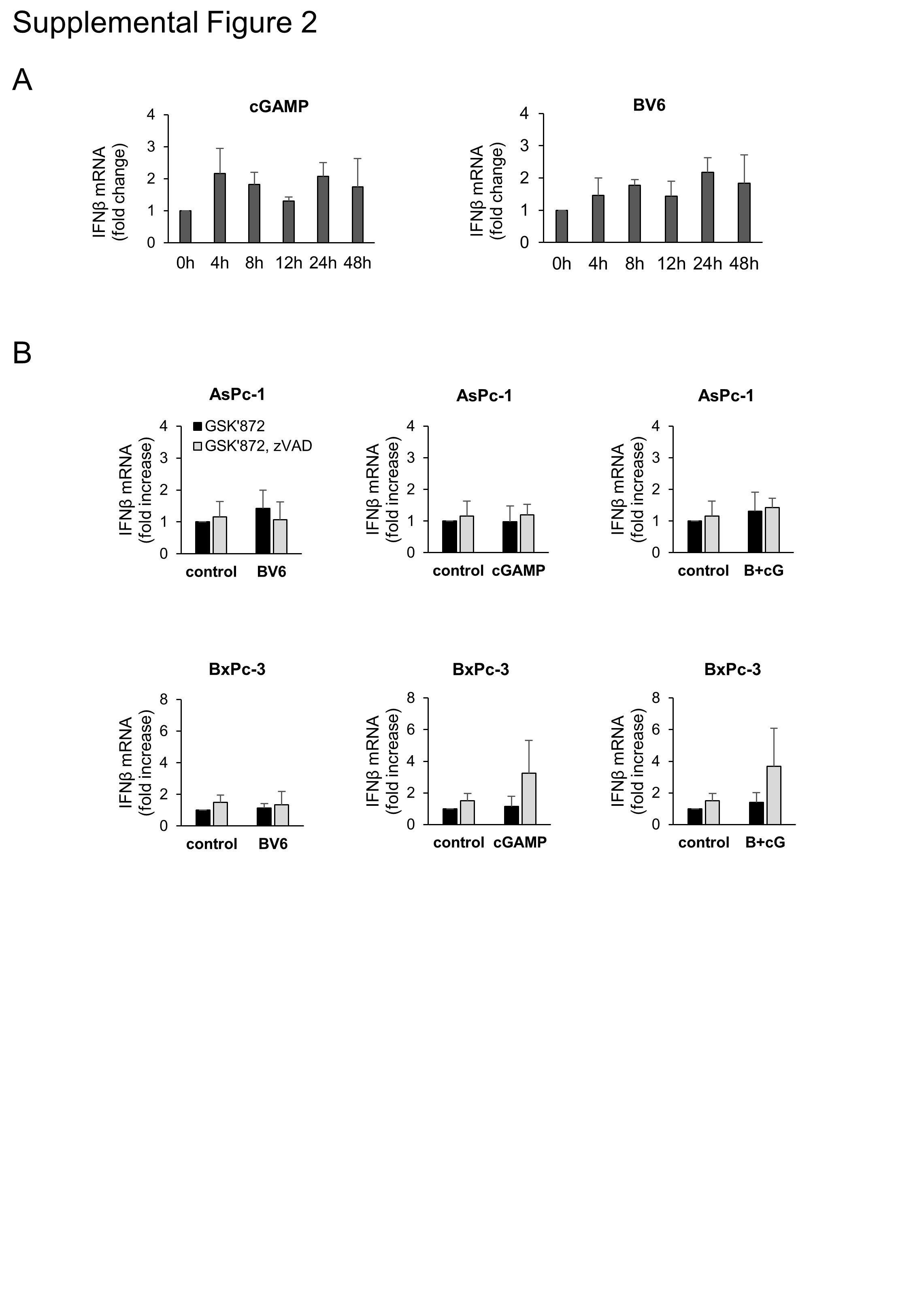

Supplement: Supplementary file 3 — Supplemental Figure 2: BV6 and 2′3′-cGAMP trigger IFN induction in PC cell lines [file 41419_2021_4014_MOESM3_ESM.tif]

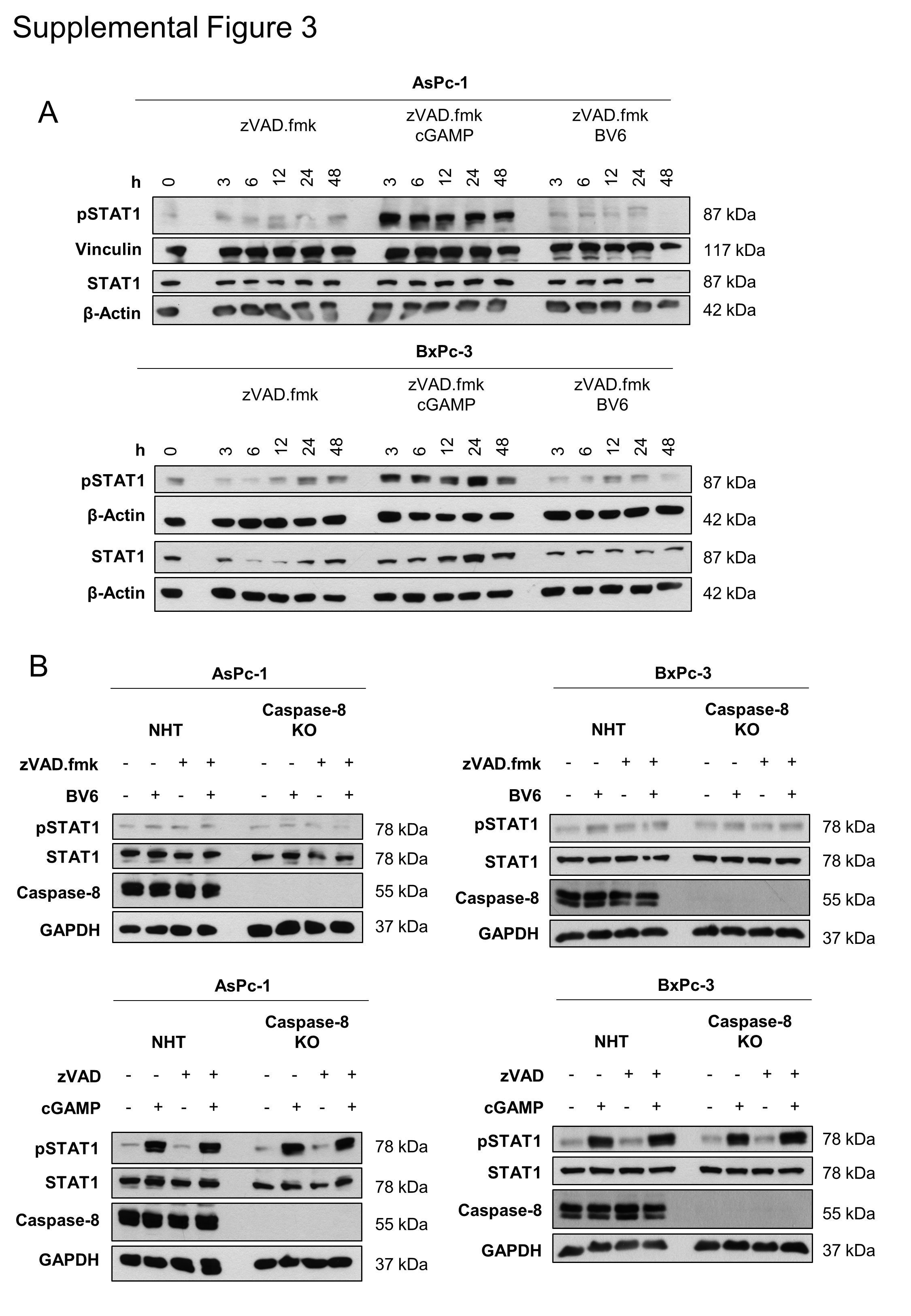

Supplement: Supplementary file 4 — Supplemental Figure 3: 2′3′-cGAMP and BV6-induce STAT1 phosphorylation in apoptosis-deficient PC cell lines [file 41419_2021_4014_MOESM4_ESM.tif]

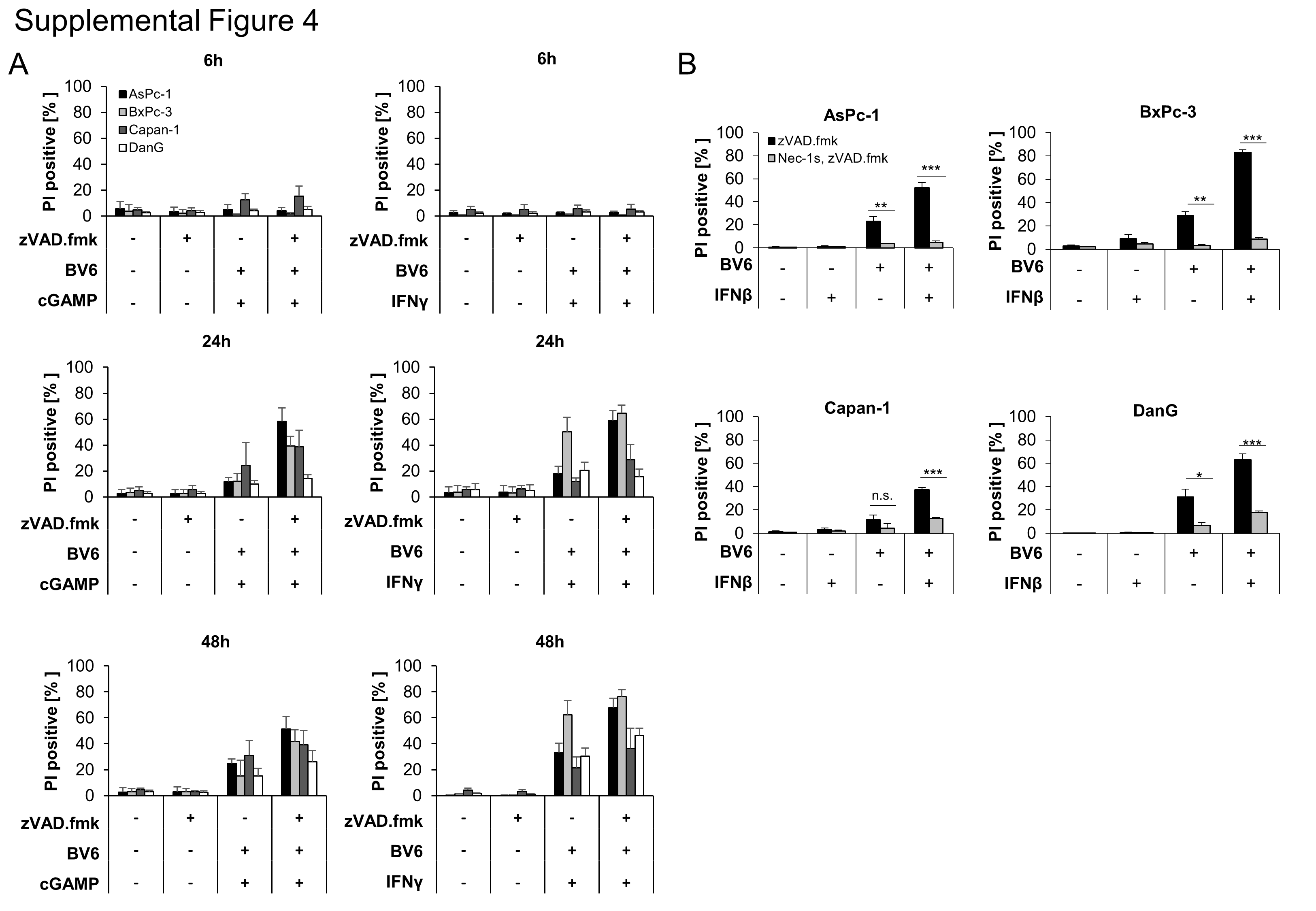

Supplement: Supplementary file 5 — Supplemental Figure 4: Time course analysis of IFN-, 2′3′-cGAMP- and BV6-induced cell death [file 41419_2021_4014_MOESM5_ESM.tif]

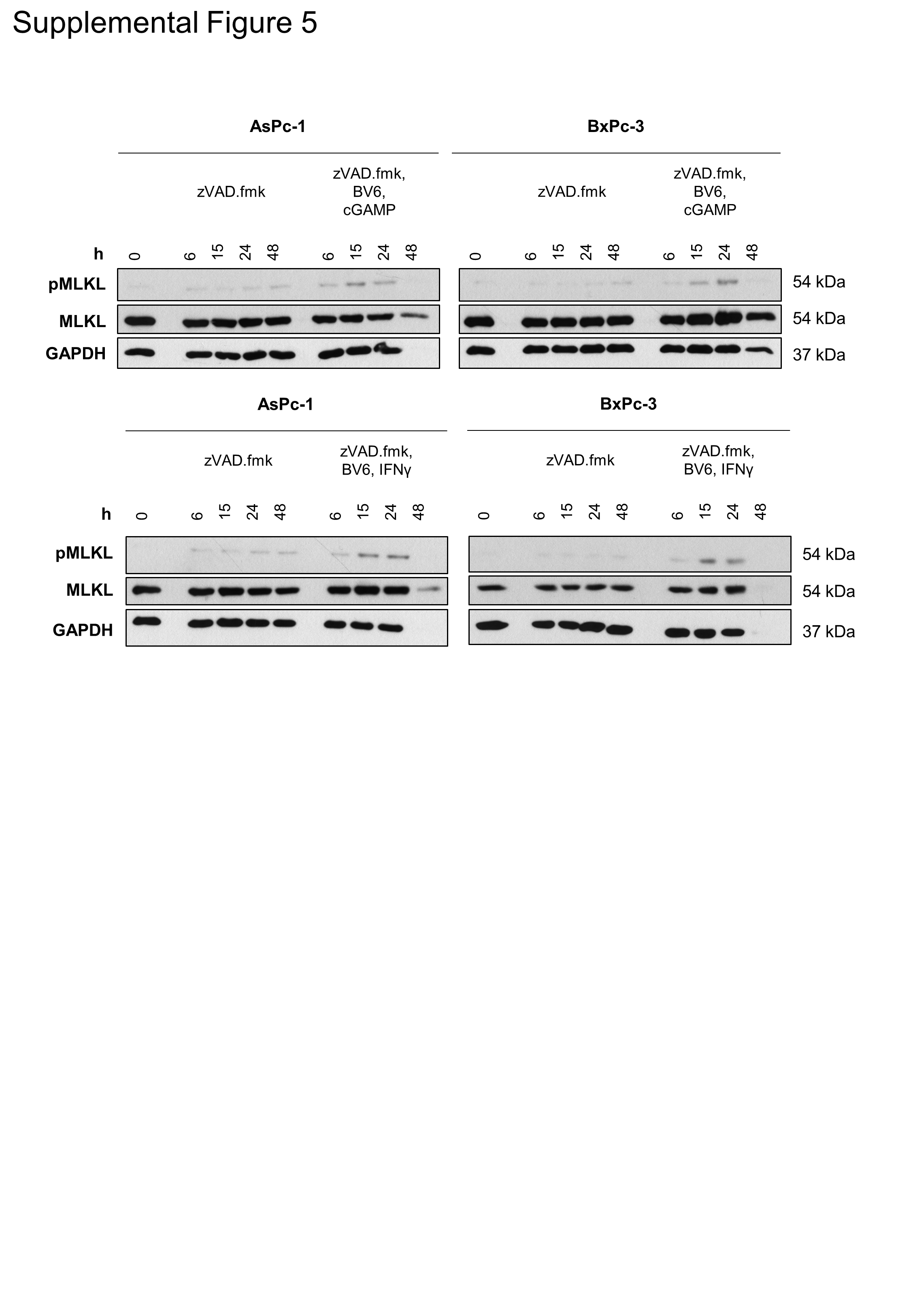

Supplement: Supplementary file 6 — Supplemental Figure 5: Time course analysis of IFNγ-, 2′3′-cGAMP- and BV6-induced MLKL phosphorylation [file 41419_2021_4014_MOESM6_ESM.tif]

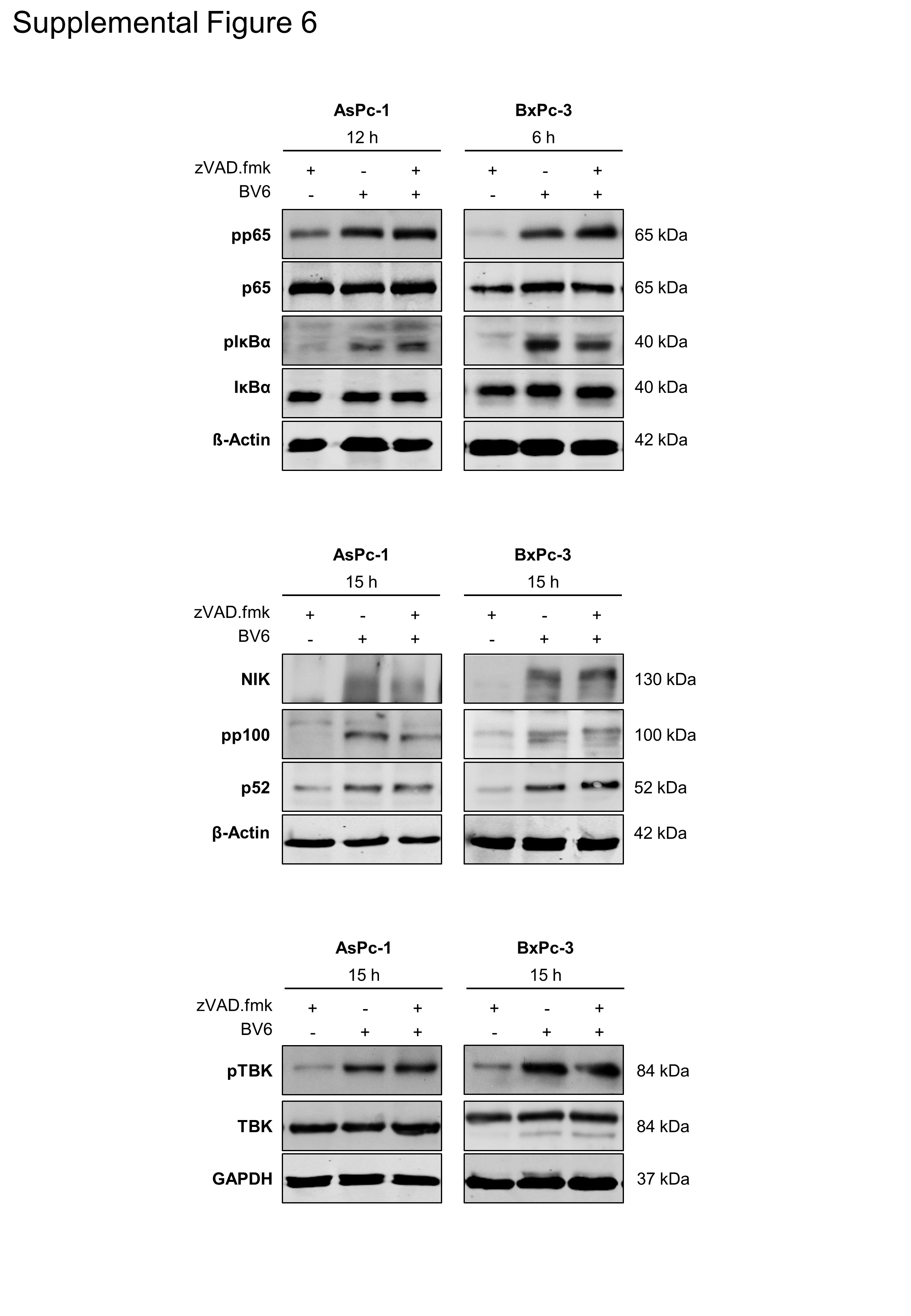

Supplement: Supplementary file 7 — Supplemental Figure 6: BV6 induces NF-κB signaling in PC cell lines [file 41419_2021_4014_MOESM7_ESM.tif]

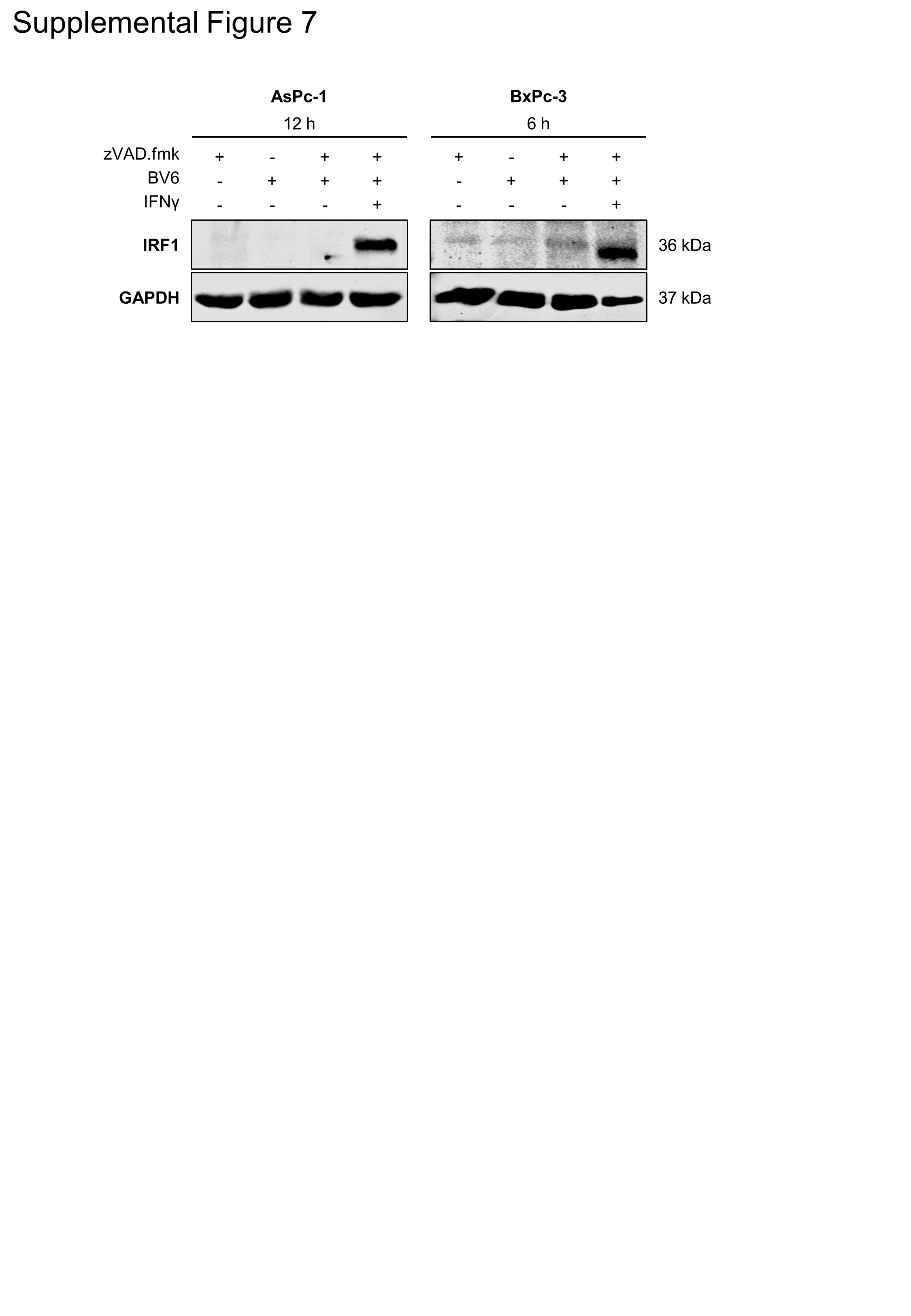

Supplement: Supplementary file 8 — Supplemental Figure 7: BV6/IFNγ/zVAD.fmk-mediated upregulation of IRF1 [file 41419_2021_4014_MOESM8_ESM.tif]
