# Supplementary material for: The Smac mimetic BV6 cooperates with STING to induce necroptosis in apoptosis-resistant pancreatic carcinoma cells
Source: Cell Death Dis. 2021 Aug 30;12(9):816. doi: 10.1038/s41419-021-04014-x (PMC8405653; doi:10.1038/s41419-021-04014-x)
Supplement: Supplementary file 9 — Supplemental Tables [file 41419_2021_4014_MOESM9_ESM.docx]

**Supplemental Table 1: Primers used in this study**

| **Target gene** | **Sequence** |
| --- | --- |
| hIRF1_for | ACAGCACCAGTGATCTGTACAAC |
| hIRF1_rev | TTCCCTTCCTCATCCTCATCT |
| IFNB1_for | ATG ACC AAC AAG TGT CTC CTC C |
| IFNB1_rev | GGA ATC CAA GCA AGT TGT AGC TC |
| hTNFα | Hs01113624_g1 |
| h28S_for | TTGAAAATCCGGGGGAGAG |
| h28S_rev | ACATTGTTCCAACATGCCAG |

**Supplemental Table 2: gRNAs used in this study**

| **Target gene** | **gRNA sequence** |
| --- | --- |
| IRF1 | #1: TTAATTCCAACCAAATCCCG |
| IRF1 | #2: TCTAGGCCGATACAAAGCAG |
| IRF1 | #3: GAACTCCCTGCCAGATATCG |
| STING | #1: CATTACAACAACCTGCTACG |
| STING | #2: GCTGGGACTGCTGTTAAACG |
| STING | #3: GCAGGCACTCAGCAGAACCA |
| Caspase-8 | #1: CACCGctacctaaacactagaaagg |
| Caspase-8 | #2: CACCGtctactgtgcagtcatcgtg |
| Caspase-8 | #3: CACCGaggggactcggagactgcga |
| NHT | #3: GGAGCGCACCATCTTCTTCA |
| NHT | #2: GGCCACAAGTTCAGCGTGTC |
| NHT | #3: GGGCGAGGAGCTGTTCACCG |
